# Anti-glucocorticoid-induced Tumor Necrosis Factor–Related Protein (GITR) Therapy Overcomes Radiation-Induced Treg Immunosuppression and Drives Abscopal Effects

**DOI:** 10.3389/fimmu.2018.02170

**Published:** 2018-09-20

**Authors:** Jonathan E. Schoenhals, Taylor R. Cushman, Hampartsoum B. Barsoumian, Ailin Li, Alexandra P. Cadena, Sharareh Niknam, Ahmed I. Younes, Mauricio da Silva Caetano, Maria Angelica Cortez, James W. Welsh

**Affiliations:** ^1^Experimental Radiation Oncology Departments, The University of Texas MD Anderson Cancer Center, Houston, TX, United States; ^2^Division of Radiation Oncology, The University of Texas MD Anderson Cancer Center, Houston, TX, United States

**Keywords:** PD1 resistance, radiotherapy, immunotherapy, GITR, cancer

## Abstract

Despite the potential to cure metastatic disease, immunotherapy on its own often fails outright or early on due to tumor immune evasion. To address this obstacle, we investigated combinations of anti-GITR, anti-PD1 and radiation therapy (XRT) in our previously developed anti-PD1 resistant 344SQ non-small cell lung adenocarcinoma preclinical tumor model. We hypothesized that targeting multiple mechanisms of immune evasion with this triple therapy would lead to an enhanced tumor-specific immune response and improve survival more so than any mono- or dual therapy. In a two tumor 344SQR murine model, treatment with anti-GITR, anti-PD1, and XRT led to significantly improved survival and an abscopal response, with half of the mice becoming tumor free. These mice showed durable response and increased CD4+ and CD8+ effector memory on tumor rechallenge. Regulatory T cells (Tregs) expressed the highest level of GITR at the tumor site and anti-GITR therapy drastically diminished Tregs at the tumor site. Anti-tumor effects were largely dependent on CD4+ T cells and partially dependent on CD8+ T cells. Anti-GITR IgG2a demonstrated superior efficacy to anti-GITR IgG1 in driving antitumor effects. Collectively, these results suggest that combinatorial strategies targeting multiple points of tumor immune evasion may lead to a robust and lasting antitumor response.

## Introduction

Lung cancer is the most common cancer worldwide, accounting for 1.69 million deaths annually (1). For patients presenting with unresectable locally advanced disease, concurrent chemoradiation is the standard treatment for those who can tolerate it. However, this treatment regimen is relatively ineffective in terms of controlling metastasis, the cause of death for most patients with lung cancer.

The benefit of radiation was previously thought to derive entirely from the reduction in tumor burden through improved local control. However, improved understanding of the role of the immune system in regulating cancer has led to the recognition that radiation therapy is a potent cause of immunogenic cell death, serving to prime the immune system with the potential to attack cancer cells outside the irradiated area. Combining immunotherapies with radiation seems to enhance systemic control, occasionally giving rise to the so-called abscopal effect (2). However, not all patients respond to immunotherapeutic agents, and even those who do often develop treatment resistance, particularly when immunotherapeutics are given as monotherapy (3, 4).

To explore the mechanisms underlying treatment resistance, our lab recently developed an anti-PD1-resistant 344SQ non-small cell lung adenocarcinoma (NSCLC) cell line that is refractory to anti-PD1 treatment (5), and with this model we found that radiotherapy helps to overcome PD1 resistance by upregulating major histocompatibility complex (MHC) class I molecules. However, even though radiation therapy can abrogate certain mechanisms of treatment resistance, it also contributes to the undesirable immunosuppressive phenotype of cancer through upregulating Tregs, both systemically and within the tumor microenvironment (6).

One of numerous target molecules currently under investigation in cancer-related immunotherapy is glucocorticoid-induced tumor necrosis factor (TNF)–related protein (GITR) (7). A member of the TNF receptor family of proteins (8), GITR was first discovered to have immunosuppressive effects when experimental elimination of GITR led to the rapid onset of autoimmune reactions (9). GITR is constitutively expressed on CD4+CD25+ regulatory T cells (Tregs) (9, 10); its low basal expression on NK cells, macrophages, dendritic cells, CD4+ and CD8+ cells increases substantially upon T-cell activation (9, 11, 12). An anti-GITR monoclonal antibody (mAb) has shown anti-tumor effects in several murine models (12, 13). In one such murine central nervous system tumor model, giving radiation therapy with anti-GITR mAb improved survival rates, although only to 24% (14). With this knowledge, we hypothesized that anti-GITR mAb therapy would synergize with radiation and anti-PD1 therapy to overcome anti-PD1 treatment resistance and improve outcomes. Hence for this study we used our anti-PD1-resistant abscopal 344SQ cancer model (5) to characterize GITR expression and determine whether combining radiation therapy with anti-GITR and anti-PD1 antibodies could elicit an abscopal response.

## Materials and methods

### Cell lines and drugs

All experiments involved using a previously derived 344SQ anti-PD1 resistant (344SQR) cell line (5). For the current study, 344SQR cells were cultured in RPMI-1640 media supplemented with 10% fetal bovine serum and penicillin/streptomycin. Agonist mouse anti-GITR IgG1 and IgG2a (clone 2D5) and anti-PD1 IgG1 mAbs (all from Bristol-Myers Squibb) were diluted in phosphate-buffered saline (PBS; pH 7.4). Other antibodies used were mouse IgG2a isotype (BE00985; BioXCell) in addition to anti-CD4 (GK1.5, 100442) and anti-CD8a (53-6.7, 100746) depleting antibodies (BioLegend).

### Tumor inoculation and treatment

**Figure 1:**
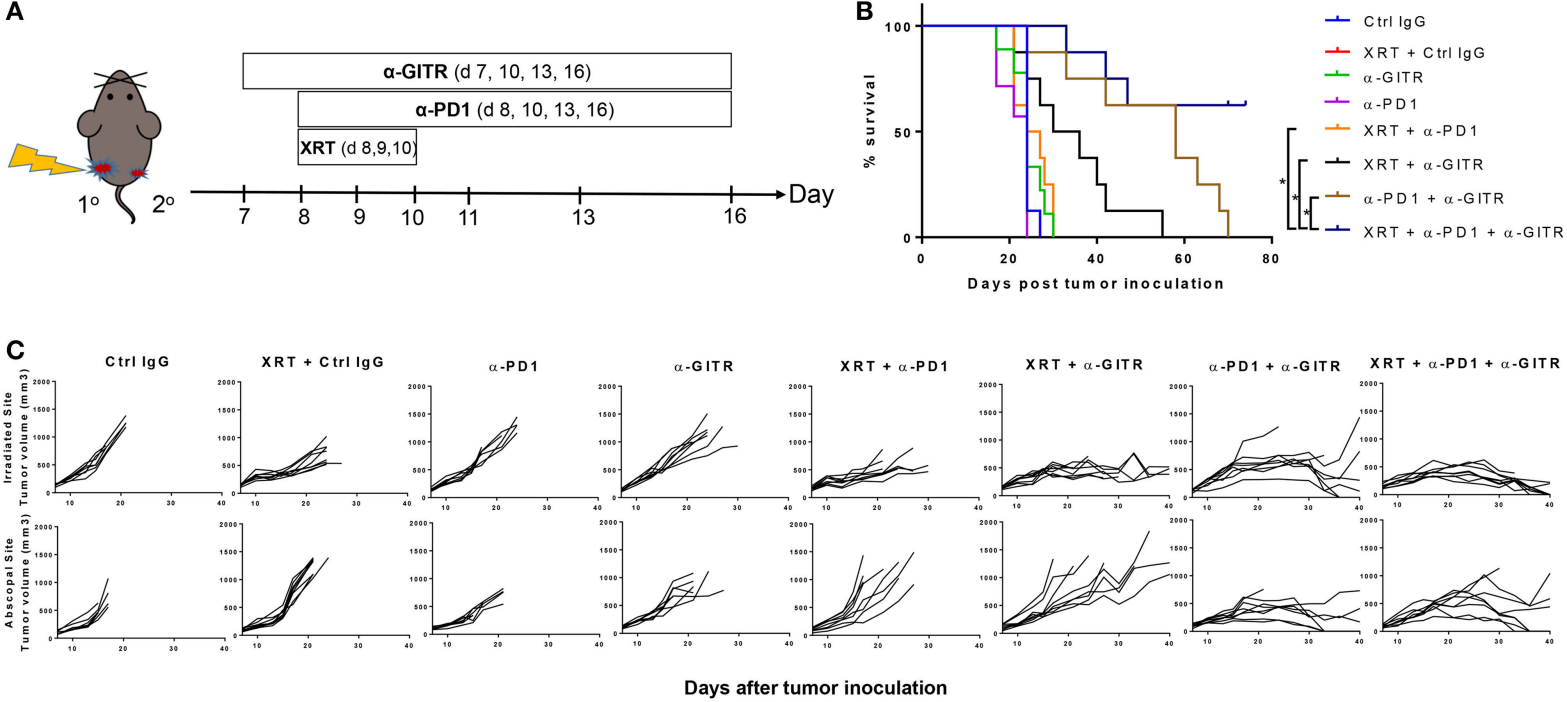
Anti-GITR combination therapy with anti-PD1 and XRT in 344SQ resistant tumors increases overall survival and promotes abscopal effects. Mice were inoculated on both right (primary) and left (secondary) legs with the second inoculation occurring 4 days after the first. **(A)** Anti-GITR therapy began on day 7 post inoculation and XRT began on day 8 post primary tumor inoculation with anti-PD1. **(B)** Plots of individual tumor growth values for primary tumors of various treatment arms (*n* = 8 per group), and **(C)** plots of survival by treatment group. *P ≤ 0.05 in Log-rank test.

For tumor inoculation, 5 × 10^5^ 344SQR cells in 50 μL PBS were injected subcutaneously (s.c.) into the right leg of 129Sv/Ev mice (male, 12–16 weeks old). Four days later, 1 × 10^5^ 344SQR cells were injected s.c. into the left leg. For tumor rechallenge experiments, 1 × 10^5^ 344SQR cells in 50 μL PBS were injected in the left leg of the tumor free mice 90 days after they became tumor free. Agonist GITR treatment or control IgG (100 μg) was given via intraperitoneal injections beginning on day 7 and continuing every 3–4 days for 4 total doses. Anti-PD1 (200 μg) was given, also by intraperitoneal injection, beginning on day 8 and continuing for 4 total doses. Radiation therapy involved three 12-Gy single fractions, given 1 day apart beginning on day 8. The timing of these treatments is shown graphically in Figure 1A. For the comparison of anti-GITR mouse IgG1 vs. mouse IgG2a, all treatments were begun 3 days later than in Figure 1A.

For depletion studies, 500 μg of anti-CD4 and anti-CD8a were given on day 6, 2 days before the radiation. Blood samples were drawn and analyzed 3 days after antibody injection to confirm immune cell depletion. After the initial bolus dose, depletion antibodies were given in 200–μg doses once a week.

Tumor lengths and widths were measured with calipers, and tumor volumes were calculated as (volume = length × width^2^/2) (5). Radiation treatments involved restraining the mice in a jig and irradiating them with a Cs-137 Suitatron IR-64 irradiator. All animal treatments and procedures were approved by The University of Texas MD Anderson Cancer Center Institutional Animal Care and Use Committee.

### Tumor immune cell isolation

Mice were killed by anesthesia and cervical dislocation at 2 days, 6 days, or 12 days after the last dose of radiation. Tumors and spleens were collected and processed as described elsewhere (1). Briefly, tumors were cut into small pieces, digested in Liberase and DNAse I for 30 min at 37°C, filtered, and separated from tumor cells by using Histopaque 1077. Spleens were homogenized and filtered and the red blood cells were lysed with ACK lysis buffer for 3–5 min. All reagents were from Sigma-Aldrich.

### Cell staining and flow cytometry

Immune cells were treated with anti-CD16/32 for 10–15 min before being stained to block Fc receptors. Cells were then stained with anti-CD45 pacific blue, anti-CD4 APC-Fire/BV510, anti-CD8 PerCP-Cy5.5, anti-GITR-APC, anti-CD39-PE, anti-OX40-PE-Cy7, anti-CD11c BV510, anti-CD11b APC-Fire, anti-CD206 PerCP-Cy5.5, anti-GITRL-PE, or anti-Gr1 PE-Cy7 (all from BioLegend) at room temperature for 20–30 min. Staining for intracellular Foxp3 (Treg cell marker) involved fixing, permeabilizing, and staining cells with an anti-Foxp3 AlexaFluor 488 for 30 min at room temperature (eBioscience). Samples were collected and analyzed on an LSRII or a Gallios (BD Biosciences) flow cytometer and analyzed with FlowJo software.

### Statistical analysis

GraphPad Prism 7.0 software was used to evaluate changes in flow cytometric and tumor growth findings. Student's *t*-tests were used to compare treatment conditions, and differences between conditions were deemed significant at *P* < 0.05.

## Results

### Anti-GITR with anti-PD1 and radiation drives abscopal effects and durable response

Anti-GITR mouse IgG2a, much like anti-CTLA4, can deplete Tregs (14), but anti-GITR is also co-stimulatory and drives T-cell proliferation and activation (15). We therefore hypothesized that combining GITR with anti-PD1 and radiation would drive strong T-cell–mediated responses in our 344SQR model through a combination of these two mechanisms. We previously showed that radiation can restore response to anti-PD1 (5) via upregulating MHC I, but we did not test this in an abscopal setting (i.e., two tumors, one in each leg). We found no significant difference in survival times among the monotherapy groups (Figure 1B). Combining anti-PD1 with radiation led to partial responses in the primary tumor, but like radiation alone, did not evoke an abscopal response in the secondary tumor, and those mice died before day 30 (Figure 1C). The combination of anti-GITR therapy with radiation (55 days), or the combination of anti-GITR with anti-PD1 (70 days), delayed primary and secondary tumor growth to a greater extent than did anti-PD1 alone (24 days), but ultimately did not produce complete responses. Only the triple combination (anti-GITR and anti-PD1 and radiation) produced complete responses, with 4 of 11 mice remaining tumor-free at 70 days post-tumor challenge.

**Figure 2:**
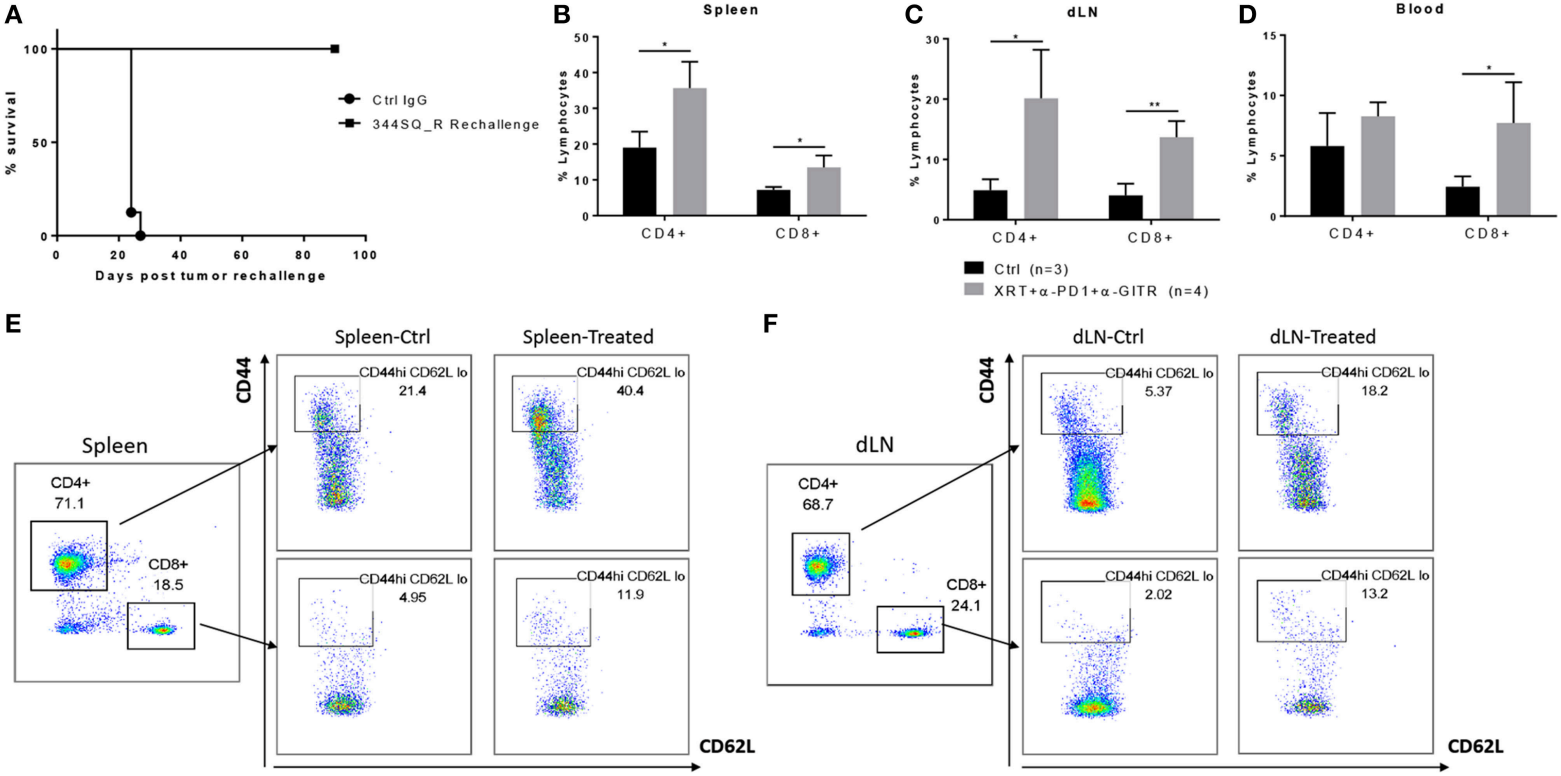
Long term effector memory is established in 129Sv/Ev mice challenged with 344SQ_R tumors and treated with XRT + anti-PD1 + anti-GITR. Mice previously treated with triple therapy who had total resolution of tumors (*n* = 4) were rechallenged with 1 × 10^6^ 344SQR cells at the abscopal site and compared with control mice (*n* = 3), prevously treated with Ctrl IgG. **(A)** Survival of rechallenged mice vs. controls. CD4 and CD8 T-cells from **(B)** spleen **(C)** secondary tumor draining lymph node and **(D)** peripheral blood are analyzed 90 days after the secondary abscopal tumor clearance. **(E, F)** Cells are gated on CD3^+^ then on CD44^hi^ and CD62L^low^ population. *P ≤ 0.05; **P ≤ 0.01 in Student *t*-tests.

GITR has been shown to expand tumor-specific CD8+ T cells and to increase effector memory, resulting in durable response and tumor elimination upon reinoculation of tumor cells (16, 17). To further investigate the mechanism of action of the triple-combination therapy (radiation + anti-PD1 + anti-GITR), these mice were rechallenged via reinoculation of 344SQ_R tumor cells 90 days after becoming tumor free. We found that the rechallenge group remained tumor free and survived (Figure 2A). Also, at 90 days post tumor rechallenge, we found that long-term effector memory (CD4+ CD44^hi^ CD62L^lo^ and CD8+ CD44^hi^ CD62L^lo^) was established in mice treated with radiation + anti-PD1 + anti-GITR and then rechallenged with 344SQ_R tumor cells. Specifically, in the spleen and secondary draining lymph nodes, the proportions of both CD4+ and CD8+ cells increased in the triple-combination-treated mice relative to the control group (Figures 2B,C); in the blood compartment, only the proportion of CD8+ cells increased in the triple-combination-treated relative to controls (Figure 2D). Gating strategy is displayed graphically in Figures 2E,F.

### Regulatory T cells express the highest levels of GITR

**Figure 3:**
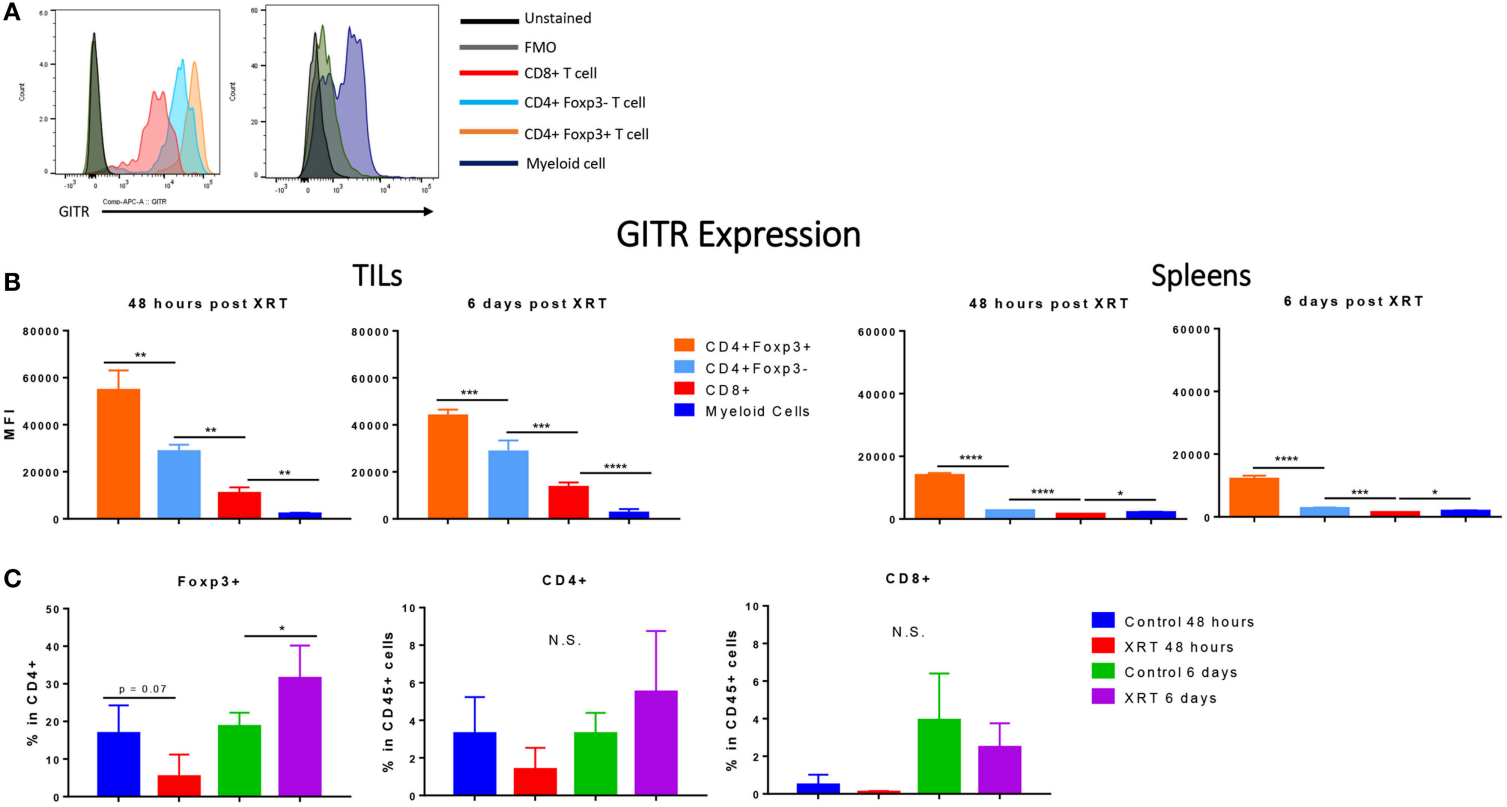
Regulatory T cells express the highest levels of GITR, especially at the tumor site, and are increased by radiation. 2 and 6 days post XRT, immune cells were isolated and phenotyped through flow cytometric analysis from either unirradiated or irradiated mice. CD45+ cells were further gated into either CD4+, CD8+ or Foxp3+ within CD4+ (*n* = 4 per group). **(A)** These values are represented graphically. **(B)** Representative MFI (mean fluorescence intensity) of GITR expression in control mice, 48 h and 6 days post XRT in CD8+, CD4+ Foxp3–, CD4+ Foxp3+, and myeloid (Supplementary Figure 1) cells with unstained and FMO controls (*n* = 16). **(C)** Averages of mean fluorescence intensity (MFI) of CD4+ Foxp3+, CD4+ Foxp3–, CD8+, and myeloid cells from all treatment groups (*n* = 18). *P ≤ 0.05; **P ≤ 0.01; ***P ≤ 0.001; ****P ≤ 0.0001 in Student *t*-tests; N.S., not statistically significant.

Immune cells at the tumor site were further analyzed for their expression of GITR. Consistent with studies from others (10, 18), we found that GITR mean fluorescence intensity (MFI) at the tumor site was highest in Tregs, followed by CD4+ and then CD8+ T cells (Figures 3A,B) (Gating strategy Supplementary Figure 1). We also detected GITR on myeloid cells, although at a lower MFI (Figure 3B). Although this trend was similar in the spleen, levels of GITR were much lower, suggesting that immune cells were in an activated state at the tumor site. Radiation did not seem to increase the expression of GITR within these subsets (data not shown). XRT led to a significant increase in the amount of Foxp3+ CD4+ T cells at 6 days compared to controls (Figure 3C).

### Anti-GITR therapy can abrogate the increase of tregs at the tumor site after radiation

**Figure 4:**
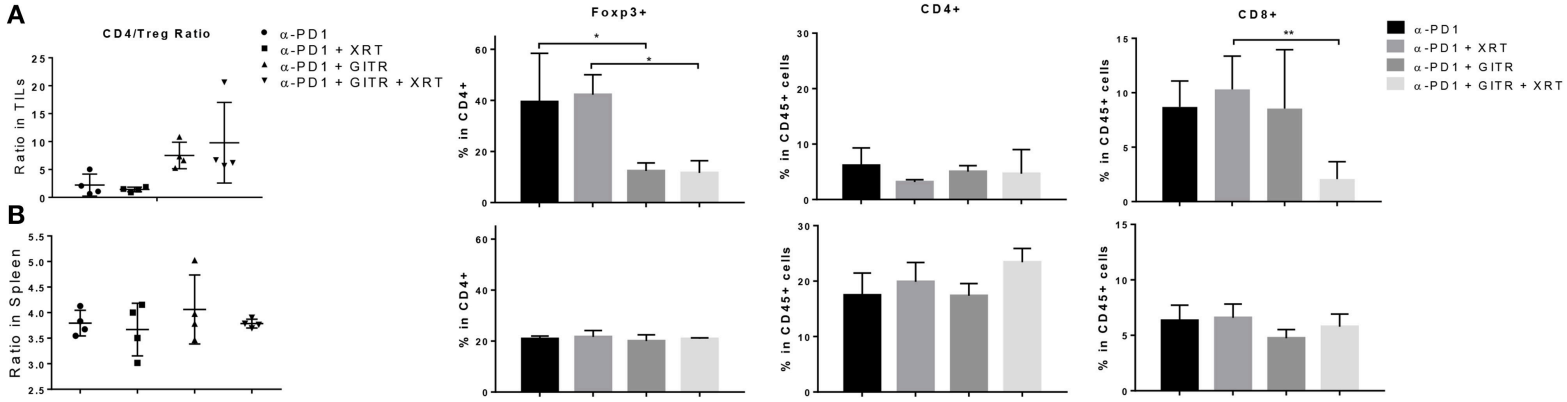
Anti-GITR therapy reduces regulatory T cells and increases the CD4 / Treg ratio at the tumor site but not in the spleen. Immune cells isolated from murine tumors were phenotyped 22 days after tumor inocluation. **(A)** Tumor-infiltrating leukocytes (TIL) and **(B)** splenocytes were isolated and evaluated for CD4, CD8, and Foxp3 expression. Foxp3+ values are shown after gating from within CD4+ cells. The CD4+ Foxp3– values were divided by the CD4+ Foxp3+ values to display the ratio. *P ≤ 0.05 in Student *t*-tests.

We proceeded to test if anti-GITR therapy was capable of depleting Tregs in 344SQR tumors. Since we had previously tested anti-PD1 in 344SQR and noted no significant effects, we decided to use PD1 as the control. In addition, we decided to test these effects at 12 days after radiation because tumor growth in the triple-combination group began to decline at that point. Interestingly, we found no significant changes in CD4 populations between the groups, but we found a decrease in CD8 T cells after the triple combination at the primary tumor site (Figure 4A). In addition, we noted that anti-GITR treatment was able to deplete Tregs at the tumor site, even in the face of increased Treg expression in the arm with RT. This in turn increased the CD4/Treg ratio. There were no significant immunological changes in the spleen (Figure 4B).

### Anti-tumor effects are largely dependent on CD4 T cells

**Figure 5:**
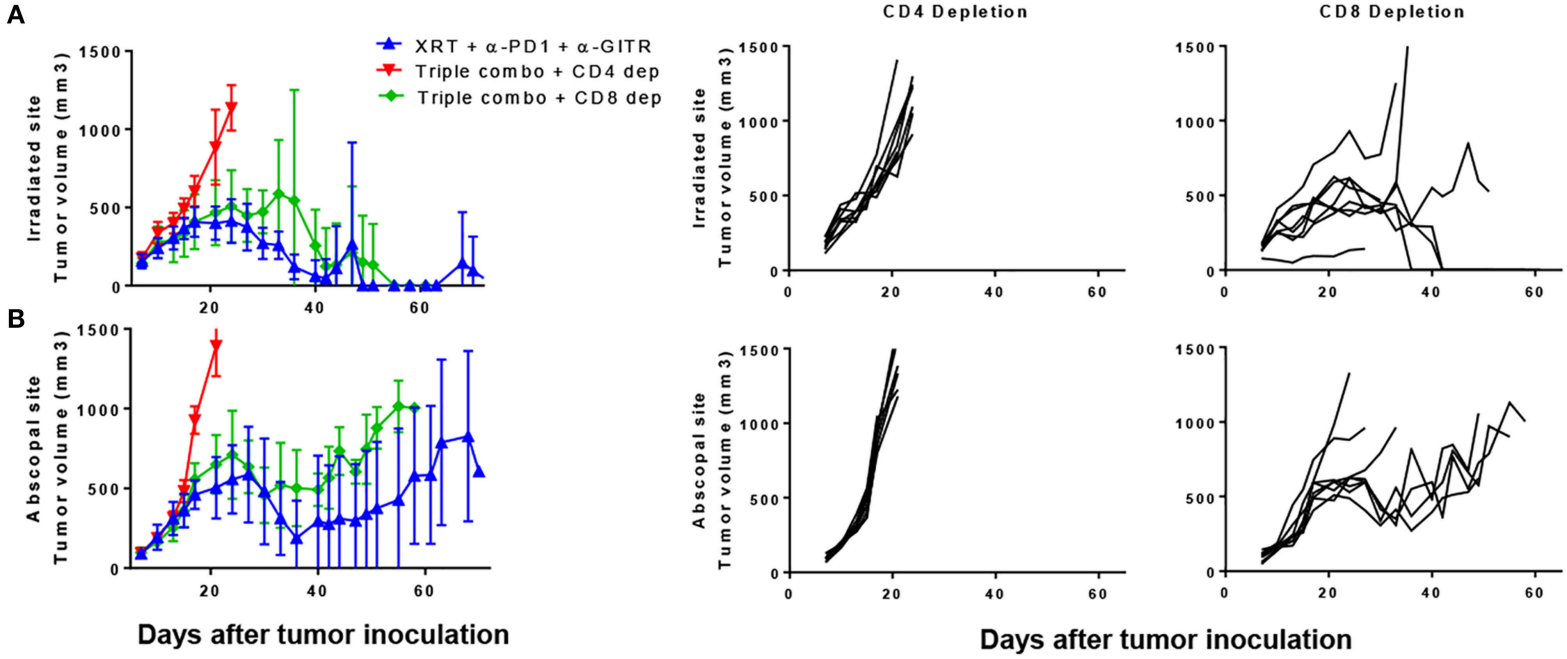
The combination of PD1, GITR, and radiation requires CD4+ T cells and partially CD8+ T cells. Mice were inoculated with primary and secondary tumors and were treated with XRT, anti-PD1, and anti-GITR, similar to previous experiments. Starting on day 6 post inoculation, depleting antibodies were administered, initially with a bolus of 500 ug and then once weekly with 200 ug. Anti-GITR therapy started on day 7 followed by XRT and PD1 on day 8. **(A)** Primary and **(B)** secondary tumors are shown from the groups (*n* = 7). Gating strategy seen in Supplementary Figure 2.

Because we noted a decrease in CD8 T cells, we tested antibody-mediated cell depletion using either anti-CD4 or anti-CD8 in the setting of the triple combination (anti-PD1, anti-GITR, and XRT). Blood was drawn 3 days after depletion and showed almost complete depletion of the respective cell populations (S3.1). We noted that the anti-tumor effects both at the primary and secondary tumors were largely dependent on CD4 T cells (Figures 5A,B). Survival was significantly shortened by anti-CD4-depletion, whereas little difference was seen between CD8-depleted and non-depleted mice after 30 days (Figure 5A). However, separation in the curves began at later time points, suggesting the requirement of CD8+ T cells in long term immune responses. This is similar to what has previously been shown with the combination of anti-GITR and XRT(14).

### Anti-GITR IgG2A is superior to anti-GITR IgG1 driving antitumor effects

**Figure 6:**
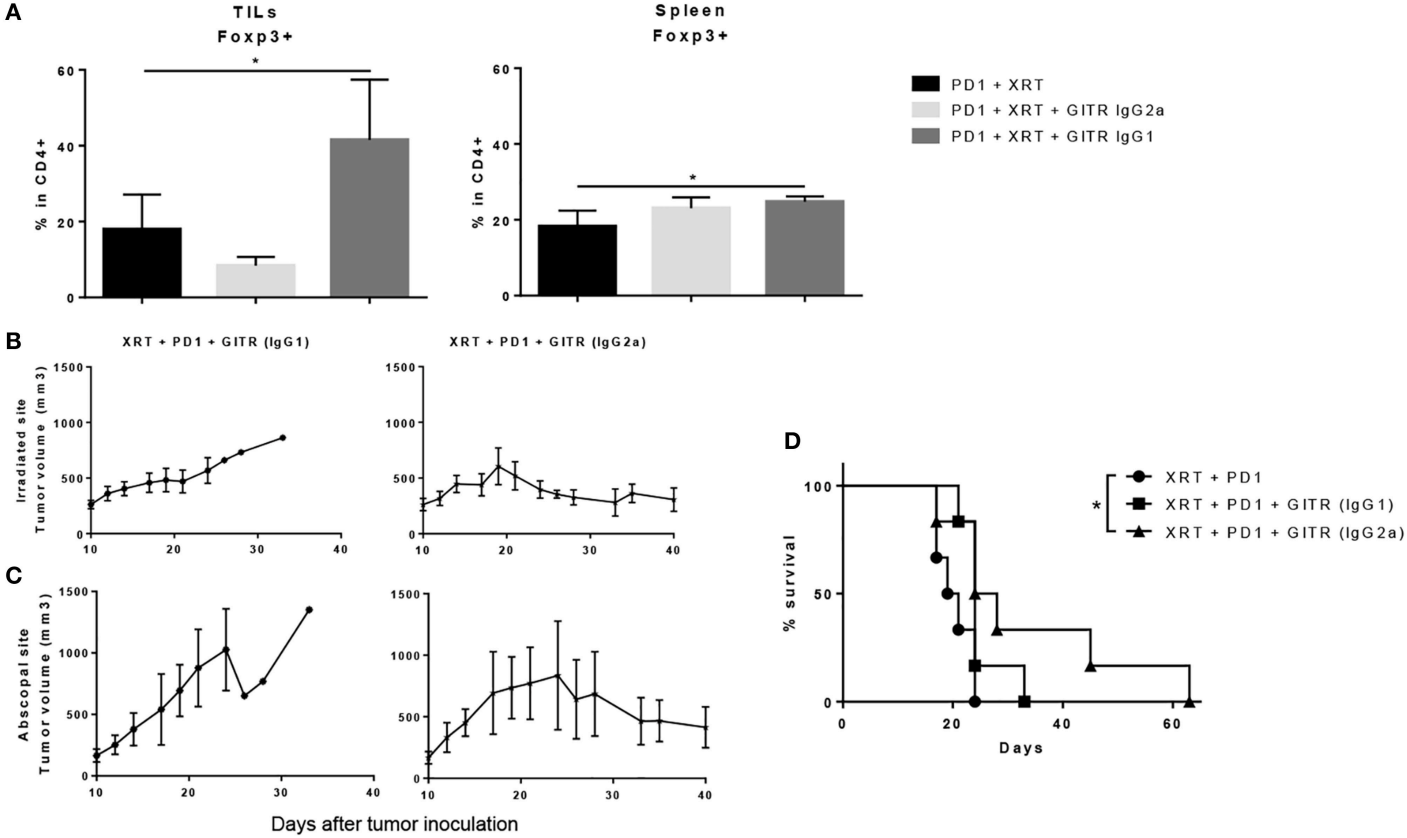
Anti-GITR IgG2a depletes Tregs at the tumor site and promotes abscopal effects when combined with radiation and anti-PD1, while anti-GITR IgG1 expands Tregs and does not promote abscopal effects. **(A)** Flow cytometry percentages of CD4+ Foxp3+ T cells in gated lymphocytes isolated from tumors and spleens, 6 days post XRT. (*n* = 6) **(B)** Primary and **(C)** secondary tumors were inoculated and XRT was delivered 10 days post tumor inoculation (*n* = 6). **(D)** Survival was monitored out to day 63 (*n* = 6). *P ≤ 0.05 in Student *t*-tests.

We also tested a non-depleting, anti-GITR mouse IgG1 isotype, known to be an agonist, to see if Treg depletion was a requirement for anti-tumor effects. When extracting tumors 6 days after the last dose of XRT, we found that GITR IgG1, rather than depleting Tregs, expanded them from a baseline of 15–20% in the CD4 T cells to around 40–45% (Figure 6A). This resulted in a drastic drop of the CD4/Treg ratio, whereas the IgG2a isotype resulted in a slight drop of Tregs as well as an increase in the CD4/Treg ratio (data not shown). The increase was not as drastic as seen previously, perhaps due to removing tumors at 6 days after radiation instead of 12 days. Again, no changes were noted in the spleen, suggesting a tumor-specific effect of both isotypes (Figure 6A).

The differences in tumor growth delay between the two isotypes were notable. We began anti-GITR treatment on day 10 and followed tumor growth once tumors were palpable. We noted that anti-GITR IgG1 did have antitumor effects at the primary site, albeit inferior to anti-GITR IgG2a (Figure 6B). However, the IgG1 isotype was incapable of driving abscopal effects at the secondary tumor site (Figure 6C). Only the anti-GITR IgG2a group, which was associated with Treg depletion, conveyed a statistically significant survival advantage when combined with XRT and anti-PD1 therapy, compared to XRT, and anti-PD1 treatment alone (Figure 6D). It is important to note that the IgG2a isotype did not drive abscopal effects in all the mice treated; this suggests that larger tumor bulk away from the primary site may impede antitumor responses.

## Discussion

Lung cancer remains the third leading cause of death worldwide (1). The majority of patients are diagnosed with progressive disease that requires more than local radiation or surgical resection. Although radiation treatment can elicit an abscopal response, it is a rare occurrence when radiation is used as a monotherapy (19). Even with combinatorial chemoradiation, the standard of care for progressive lung cancers, systemic responses are rare and long-term survival remains low. Immunotherapeutic targets such as PD1 and CTLA4 have been proven as efficacious in clinical trials (4, 20). Despite their significant potential, the majority of patients are resistant to immunotherapy. As a result, there is a rapidly growing body of research looking at combinatorial therapies to enhance the effects of immunotherapy, particularly in patients who prove resistant to monotherapy. Preclinical and clinical evidence suggests radiation and immunotherapies are synergistic in the treatment of non-small cell lung cancer and other solid tumors (4, 5, 21, 22). We have only just begun to identify mechanisms which attribute to the synergistic effects.

Our previous study elucidated one way in which radiation therapy compliments immunotherapy and may help overcome treatment resistance. First, we developed an anti-PD1 resistant murine model. Then, we demonstrated that XRT mediated type I IFN-dependent upregulation of MHC I on tumor cells, resulting in resensitization of tumors to anti-PD1 treatment (5). Although the combination of XRT and anti-PD1 therapy ameliorated response rates, abscopal responses were still low overall, perhaps due to the inability of radiation to upregulate MHC I at distant sites. In this study, we sought to improve abscopal response rates. The addition of the Treg depleting anti-GITR IgG2a resulted in enhanced abscopal effects and even complete tumor regression at both primary and secondary tumor sites. Furthermore, XRT, anti-PD1 and anti-GITR IgG2a agonists were all required to drive durable responses. We believe the triple combination therapy is necessary to elicit lasting systemic reduction or elimination of tumors for the reasons we discuss below.

XRT alone has long been noted to affect changes in immune profiles, specifically with regards to the radioresistance of Tregs (6). While Tregs are relatively more radioresistant than other lymphocytes, they still decrease after radiation doses. We demonstrated that at high doses of 12 Gy x 3, Tregs are decreased proportionally after 2 days with the other immune cells profiled. This suggests that there may be a threshold level of radiation dose where this relative radioresistance diminishes. Cao and colleagues noted similar effects with increasing radiation doses with human Tregs *in vitro* (23). Even though we noted Tregs were decreased shortly after XRT, they returned at significantly higher percentages by 6 days post XRT compared to non-irradiated mice. TGF-β (24, 25) and the CCL17,22/CCR4 (26) axis may be playing a role in this upregulation of Tregs after XRT, although it is unclear if this is due to Tregs that survived the initial doses of XRT or their recruitment to the tumor site after XRT.

The Tregs at the tumor site expressed the highest levels of GITR. Likely the major contributor to the benefits of the addition of anti-GITR therapy to XRT and anti-PD1 in our experiments, other research has highlighted the ability of anti-GITR antibodies to deplete Treg cells and also perhaps cause T effector cells to be less suppressed by active Treg cells (27). Further confirming the role of GITR in the regulation of Tregs, it has been shown that GITR-/- mice have demonstrated similar percentage of T cell subtypes with the exception of significantly decreased CD4+CD25+ Tregs (11).

Aside from decreased Tregs, anti-GITR therapy improved survival via promotion of durable, tumor-specific memory in our study. Previous studies have demonstrated the ability of anti-GITR agonist mAb to expand tumor-specific CD8+ effector memory. Our study similarly demonstrates that GITR plays a role in the expansion of both CD4+ and CD8+ effector memory. In line with our findings, researchers reported, in a phase 1/2 clinical trial combining anti-PD1 and anti-GITR mAbs for patients with advanced solid tumors, the dual immunotherapy was associated with an increased proliferation and activation of CD4+ and CD8+ effector and central memory cells (7). It is unclear whether the CD4+ effector memory was directly expanded by GITR therapy, or if it was the indirect result of GITR-induced CD8+ T cell expansion. Considering we saw significantly greater tumor growth in our CD4+ vs. CD8+ depletion studies, we suspect the mechanism is likely the former.

Interestingly, administration of the anti-GITR IgG2a antibody only in combination with XRT and anti-PD1 displayed reduced CD8+ T cells. This led us to hypothesize that our effects were CD4+ Foxp3– T cell-mediated. Upon administering CD4+ depleting antibodies, mice displayed minimal responses, similar to control groups. Neither IgG2a nor IgG1 expanded CD4+ or CD8+ T cells within TILs or the spleen (Supplementary Figure 3). Other groups have had similar findings with regards to Treg depletion and radiation. Bos and colleagues utilized Foxp3–DTR mice given mammary tumors (28). Administration of diptheria toxin in these mice results in depletion of Tregs, and when combined with radiation, resulted in enhanced tumor growth delay. While they did not specifically deplete CD4+ T cells in combination with XRT and diptheria toxin, they did deplete the CD4+ T cells after diptheria toxin alone. This depletion resulted in similar tumor growth to the untreated group. Similar CD4+ T cell-driven effects were noted by Patel et al. in their study with GITR agonism in combination with XRT for brain tumors (14). In contrast to their findings, we saw superior effects with the IgG2a isotype rather than the IgG1 isotype. As noted in their study, this may be due to the differences in Treg depletion with IgG2a isotypes when passing the blood-brain barrier. We also observed that administration of anti-GITR IgG1 agonist resulted in expansion, rather than depletion, of Tregs. This may be due to differences in Treg recruitment through the blood brain barrier.

While our results demonstrate significant potential to minimize or even eliminate systemic disease, likely through enhanced abscopal responses, it is important to recognize the differences in GITR expression in mice compared to humans. In murine models, there is low basal GITR expression in CD4+ and CD8+ T cells (11, 29). Recent work has also highlighted expression of GITR in murine NK cells, NKT cells, B cells, and some monocytes and granulocytes (22). Critical to our study, GITR is constitutively expressed on T regulatory cells. Further studies by Ronchetti et al. also suggest that mouse GITR may be a co-stimulatory signal in the activation of CD8 T effector cells (30), which is in line with our results demonstrating a negative effect of CD8+ T cell depletion, although the depletion did not fully abrogate the decreased tumor growth seen in mice treated with anti-GITR, anti-PD1, and radiation therapy.

There are several key differences in expression of GITR in humans. Structurally, human GITR demonstrates trimeric binding to GITR-L, while their murine counterparts demonstrate dimeric binding (29). Human GITR is not expressed on CD4+ and CD8+ T cells peripherally, but expression does dramatically increase similar to murine models upon activation (31, 32). The major distinction in human vs. murine GITR is that human GITR is not constitutively expressed on all Treg cells. Instead, it is expressed at low basal levels in CD4+ CD25+ and CD25- cells, as well as CD8+CD25+ Tregs (32, 33). A more recent study detected the highest levels of GITR in human peripheral CD4+ Tregs, T effector memory cells, and TH17 cells (22). Within the tumor microenvironment, human GITR is expressed on approximately 31 and 11% of CD4+ and CD8+ TILs, compared to nearly 100% of both cell types in murine tumor models (22). However, comparing murine vs. human Tregs within TILs revealed nearly identical frequency and intensity of expression. Although the effects of anti-GITR mAb therapy on each immune cell type have yet to be fully elucidated, it is clear that such treatment suppresses the inhibitory T regulatory cells within the tumor microenvironment and is most likely the driver of synergism between XRT and anti-GITR mAb therapy in both mouse tumor models and potentially humans, as evidenced by the ongoing clinical trial investigating anti-GITR and anti-PD1 therapies (7). At the time this paper was written, to translate our work into humans, we are in the process of amending an ongoing clinical trial to combine anti-GITR with anti-PD1 and stereotactic radiation.

In conclusion, these findings indicate the necessity for targeting multiple mechanisms of the immune system to avoid tumor immune escape. This study proposes that anti-GITR therapy in combination with anti-PD1 and XRT may be beneficial to those who are no longer responsive to anti-PD1 therapy. While Tregs may not be the main perpetrators in generating resistance to anti-PD1, they do appear to play a prominent role in the immune response following XRT. Through targeting this population with anti-GITR and/or other drugs, immune responses directed by CD4+ and CD8+ Foxp3– T cells may be sufficient to drive long-term responses. Future studies should seek to identify mechanisms of treatment resistance to allow for more personalized and effective treatments.

## Author contributions

All authors contributed to experimental data. TC and JS wrote the first draft of the manuscript and all authors contributed to revisions and edits.

### Conflict of interest statement

JW reports support from University of Texas MD Anderson, Healios, MolecularMatch, OncoResponse; non-financial support from Reflexion Medical, Checkmate Pharmaceuticals, grants from BMS, Merck, Varian; lab research support from Incyte, Merck, Calithera, Checkmate Pharmaceuticals, Mavu, Nanobiotix and Aileron; and grants from OncoResponse. The remaining authors declare that the research was conducted in the absence of any commercial or financial relationships that could be construed as a potential conflict of interest.
