# Supplementary material for: Anti-glucocorticoid-induced Tumor Necrosis Factor–Related Protein (GITR) Therapy Overcomes Radiation-Induced Treg Immunosuppression and Drives Abscopal Effects
Source: Front Immunol. 2018 Sep 20;9:2170. doi: 10.3389/fimmu.2018.02170 (PMC6158365; doi:10.3389/fimmu.2018.02170)
Supplement: Supplementary file 1 [file Image_1.pdf]

Supplementary Figure S1

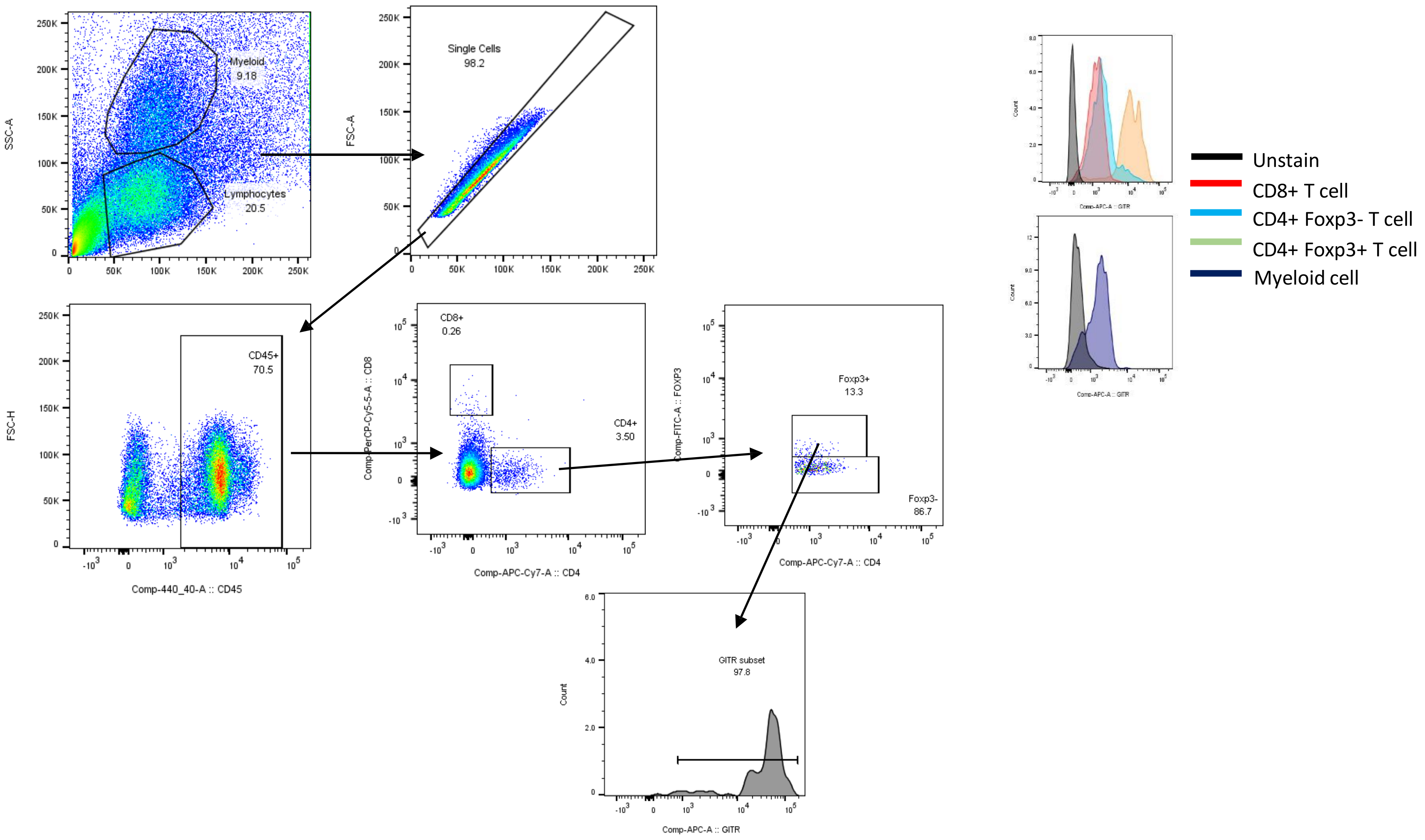

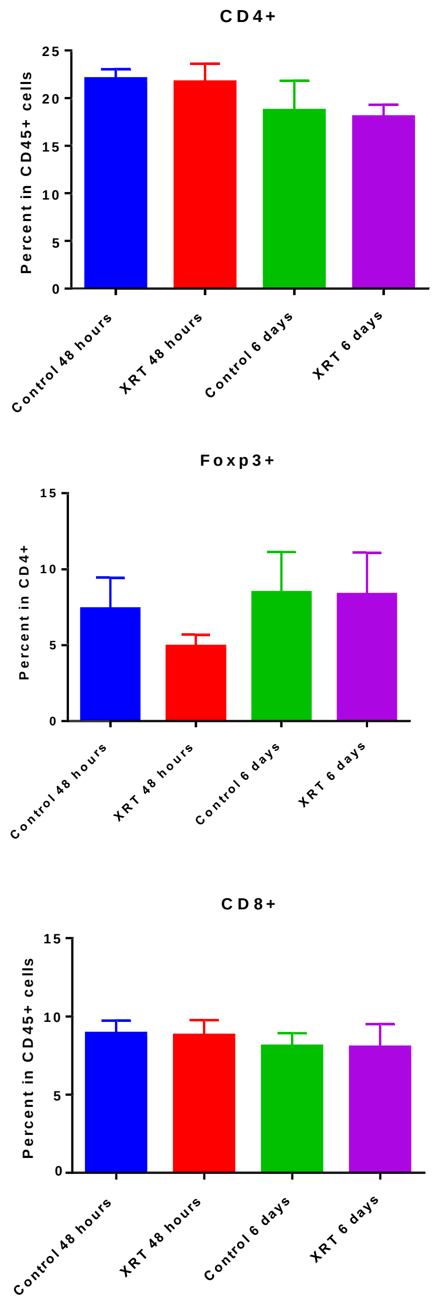

Supplementary Figure 1. Gating strategy, and representative mean fluorescence intensity (MFI) in spleen. Cells were gated on either lymphocytes or myeloid populations, followed by selection of singlet cells. Gate was then placed on CD45^{+} population followed by CD4 and CD8. Next, cells were gated on CD4^{+} population to obtain Foxp3^{+} cell percentages.

Supplementary Figure S2

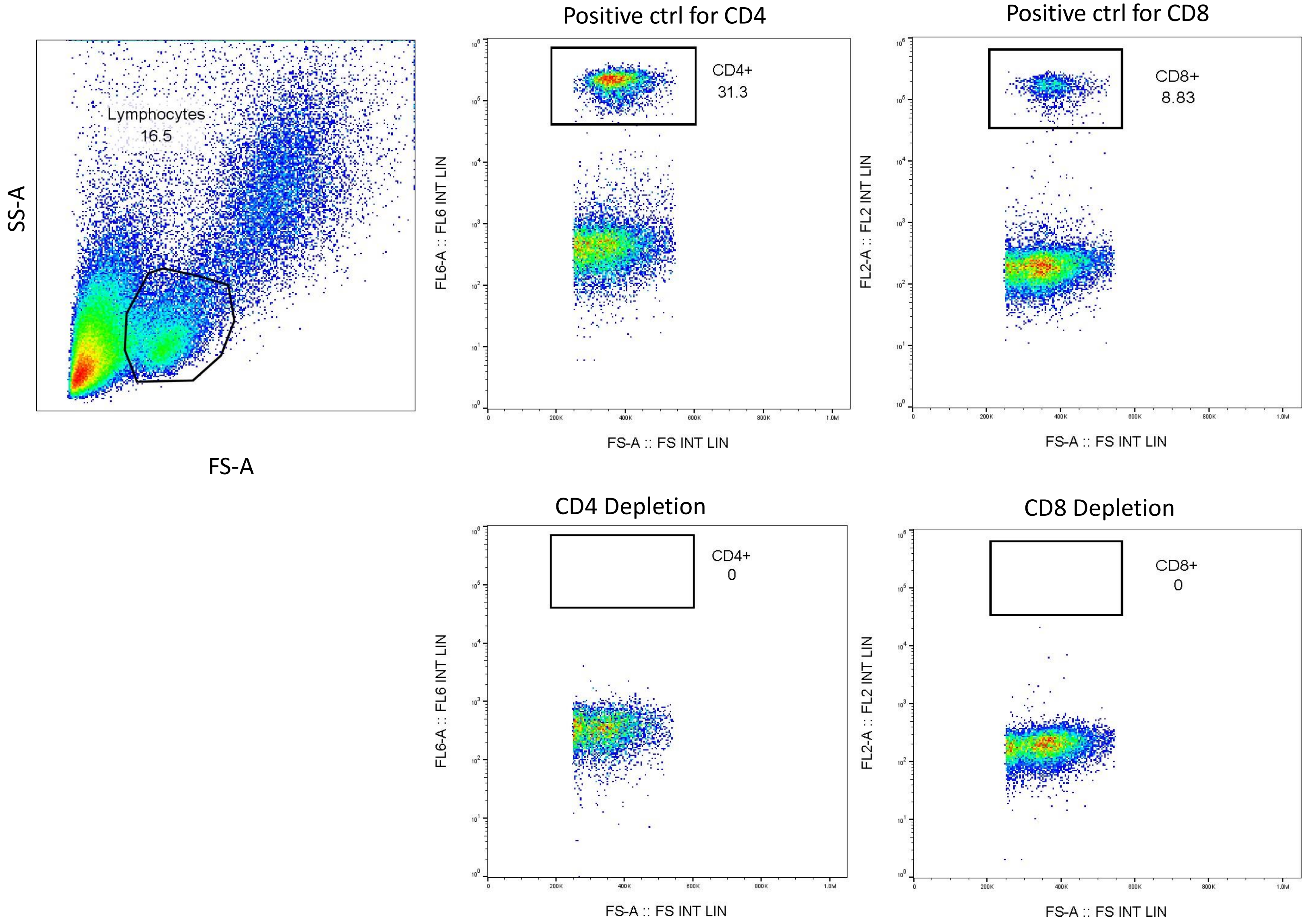

Supplementary Figure 2. Representative flow cytometric analysis showing CD4 and CD8 depletion. Mice were injected with either anti-CD4 or anti-CD8 depleting antibodies (500 µg/inj., i.p.) before treatment. Blood was collected and phenotyped 3 days after depletion using CD4-APC and CD8-PE cell surface markers.

Supplementary Figure S3

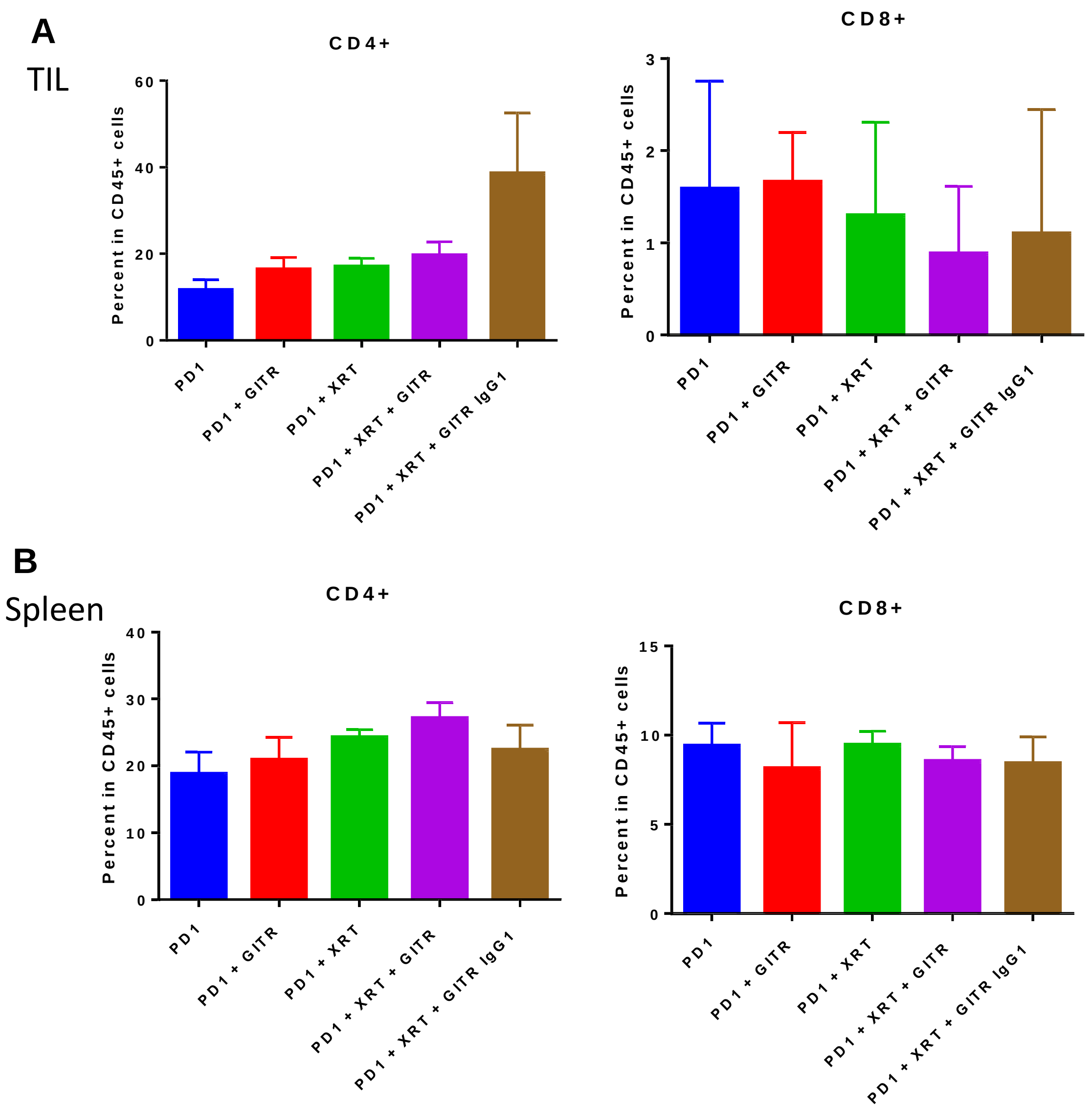

Supplementary Figure 3. Neither IgG2a nor IgG1 expand CD4 or CD8 T cells within TILs or the spleen. Flow cytometry percentages of CD4+ T cells in gated lymphocytes isolated from (A) tumors and (B) spleens, 6 days post XRT.
